# Short Amplexus Duration in a Territorial Anuran: A Possible Adaptation in Response to Male-Male Competition

**DOI:** 10.1371/journal.pone.0083116

**Published:** 2013-12-10

**Authors:** Ming-Feng Chuang, Mark A. Bee, Yeong-Choy Kam

**Affiliations:** 1 Department of Life Science, Tunghai University, Taichung, Taiwan; 2 Deparment of Ecology, Evolution and Behavior, University of Minnesota, Twin Cities, St. Paul, Minnesota, United States of America; University of Windsor, Canada

## Abstract

Mating duration is a reproductive behaviour that can impact fertilization efficiency and offspring number. Previous studies of factors influencing the evolution of mating duration have focused on the potential role of internal sperm competition as an underlying source of selection; most of these studies have been on invertebrates. For vertebrates with external fertilization, such as fishes and frogs, the sources of selection acting on mating duration remain largely unknown due, in part, to the difficulty of observing complete mating behaviours in natural conditions. In this field study, we monitored breeding activity in a population of the territorial olive frog, *Rana adenopleura*, to identify factors that affect the duration of amplexus. Compared with most other frogs, amplexus was short, lasting less than 11 min on average, which included about 8 min of pre-oviposition activity followed by 3 min of oviposition. We evaluated the relationship between amplexus duration and seven variables: male body size, male condition, operational sex ratio (OSR), population size, clutch size, territory size, and the coverage of submerged vegetation in a male’s territory. We also investigated the influence of these same variables, along with amplexus duration, on fertilization rate. Amplexus duration was positively related with clutch size and the degree of male-bias in the nightly OSR. Fertilization rate was directly related to male body size and inversely related to amplexus duration. Agonistic interactions between males in amplexus and intruding, unpaired males were frequent. These interactions often resulted in mating failure, prolonged amplexus duration, and reduced fertilization rates. Together, the pattern of our findings indicates short amplexus duration in this species may be an adaptive reproductive strategy whereby males attempt to reduce the risks of mating and fertilization failures and territory loss resulting from male-male competition.

## Introduction

Sexual selection is an important evolutionary force that favours phenotypic and behavioural characteristics that enhance reproductive success [1]. One important reproductive behaviour under the influence of sexual selection is the length of time males and females spend mating [2]. Previous studies have revealed positive relationships between mating duration and offspring number and fertilization efficiency in mammals [3,4], birds [5–7], lizards [8], fishes [9], arachnids [10,11], and insects [12–15]. Therefore, mating duration is a functional behaviour that can influence reproductive success.

Numerous factors influence mating duration across diverse taxa, including body condition [13,16–19], sex ratio [15,16,20–22], population density [15,23], male mating strategy [14,24], female mating history [11,17,24–27], clutch size [19,28,29], mating location [30], and sexual conflict [12,31]. However, our understanding of causal factors affecting mating duration is based almost exclusively on studies of animals with internal fertilization, particularly of insects. For example, mating duration in insects can be prolonged by mate-guarding behaviours that reduce the intensity of sperm competition [15,21]. Moreover, male insects can prolong mating duration depending on the mating history of females, for example, to remove sperm from other males or increase sperm loading to ensure their success in sperm competition [17,25]. In contrast to internal fertilizers, animals with external fertilization do not store sperm and both eggs and sperm are released simultaneously or sequentially into the environment. Thus, factors influencing variability in mating duration in external fertilizers could well be different than those operating in internal fertilizers. 

External fertilization is a common reproductive mode among oviparous animals, and most species of anuran amphibians fertilize eggs externally. Typically, male frogs clasp females dorsally and position themselves so as to bring their cloacae in close proximity before coordinated gamete release, a mating posture known as “amplexus” [32,33]. Amplexus can be divided into three phases [34,35]. During a pre-oviposition period, the amplectant female may search for suitable oviposition sites or the pair may cooperate to prepare a burrow or nest of some kind. Eggs are released and fertilized during the oviposition period (“oviposition time” of [33]). Finally, some species also have a post-oviposition period, which may function to allow amplectant pairs to ensure complete fertilization of eggs [34,35]. The total amount of time that pairs spend in amplexus (defined as the sum of the durations of all three periods; hereafter, “amplexus duration”) varies greatly among anuran species and can last from less than one hour to more than one month (reviewed in: [33,35]). Qualitative evidence suggests several factors contribute to variation in amplexus duration among anurans, including time spent searching for or preparing nests [34,36], egg laying patterns (e.g., single versus multiple bouts of egg laying), types of egg clutch (e.g., egg string, foam nest, or egg mass) [33], and male-male competition [35,37]. However, we still lack detailed quantitative studies of the factors that influence amplexus duration in anurans. The general difficulty of observing entire amplexus events under field conditions, combined with the sensitivity of amplexus behaviour to disturbance, have hindered research aimed at uncovering the factors influencing variation in amplexus duration [33,36,38]. 

Here, we report results from a field study of the Southeast Asian olive frog, *Rana adenopleura* (Ranidae), in which we investigated potential causes and consequences of various factors influencing amplexus duration. The olive frog occurs widely throughout Southeast Asia and is common in the lowland mountain areas of subtropical China and Taiwan [39]. Adult frogs breed in ponds, lakes, and slowly flowing streams from March to October [40]. Males are territorial, usually call from dense vegetation, and defend their territory using vocalizations, physical fights, or both [41]. During the breeding season, mature males can be identified easily using several secondary sex traits, including a pair of vocal sacs and a pair of yellow-orange shoulder glands on the lateral abdomen.

During preliminary work on this species, we made several key observations of entire amplexus events that allowed us to identify likely physical, behavioural and ecological factors that might influence amplexus duration in this species. To initiate amplexus, females approached and contacted calling territorial males, at which time the males clasped the females to form amplectant pairs. Before oviposition, amplectant pairs moved to an oviposition site within the chosen male’s territory that contained submerged aquatic grass. During the pre-oviposition period, the amplectant female slowly and repeatedly turned in place, producing a basin-like depression in this aquatic vegetation where it later deposited eggs. Females laid their eggs in a large mass during a single bout, as reported for some other ranid frogs [42,43]. Importantly, agonistic encounters during amplexus events were frequent. As in some other frogs [35,37,44], unpaired males frequently attacked amplectant pairs. These observations suggested reproductive interference via direct male-male competition might also have important influences on amplexus duration in this species. Based on these initial observations, we conducted a more thorough study of amplexus events and used generalized linear mixed-effect models to describe the relationships between amplexus duration, fertilization rate, and the following seven factors: male body size, male condition, operational sex ratio (OSR), population size, clutch size, territory size, and the coverage of submerged vegetation in a male’s territory. 

## Materials and Methods

### Study site

We conducted field observations in a natural forest at the Lien-Hua-Chih Research Center (576 to 925 m in elevation) (120°52’59.5’’E, 23°55’8.9’’N) in central Taiwan. Lien-Hua-Chih receives approximately 2,600 mm of rainfall annually, approximately 80% of which falls during the wet season lasting from May through September, when local thunderstorms and typhoons bring copious amounts of rain. The mean annual temperature at the site is 20°C, and the maximum and minimum average monthly temperatures occur in July (24°C) and January (14°C), respectively. We monitored a population of *R. adenopleura* in a permanent pond (5 m x 10 m, maximum depth of approximately 1 m in the centre). Submerged aquatic grass, *Blyxa echinosperma*, was the most abundant species of vegetation in the pond and was also the most common oviposition substrate used by females. A paved walkway around approximately 50% of the pond’s perimeter provided easy accessibility for capturing and viewing frogs throughout the pond under the low light levels provided by headlamps (about 50 lumen for monitoring their behaviour and 15 lumen for marking animals). 

### Field protocol

Between late June and early September, 2008, we visited the breeding pond on 68 consecutive nights. On four of these nights, observations could not be made because of heavy rain and flooding associated with two different typhoons, yielding a final sample of 64 observation nights. We censused the pond between three and eight times on each observation night. Before starting the study, we built a 1 m × 1 m grid system from vertical PVC pipes spaced every 1 m in all directions across the entire pond. We used this grid to collect two types of data. First, we used it to pinpoint the location in the grid system (to the nearest decimetre) of each frog observed during a census as well as the positions of egg masses laid during observed amplexus events. Second, at the approximate midpoint of the study (on nights 44-45 of 68), we used it to map the coverage of submerged vegetation throughout the entire pond onto A3 graph paper. Vegetation coverage did not change appreciably over the 68 observation days. At the completion of the field observations, we mapped male territories (see below) onto the spatial distributions of both vegetation, to compute the proportion of each resident male’s territory with submerged vegetation, and the locations of oviposited egg masses, to assess whether females laid eggs in males’ territories. 

During each census, we walked slowly around the perimeter of the breeding pond, determined all frogs present, and recorded their position in the grid as well as any calling behaviour we observed. The first time frogs were encountered during a census, they were captured, measured (SVL, snout-vent length, to the nearest 0.01 mm) and weighed (to the nearest 0.05 g). We used linear regression to compute the residuals of a regression of body mass on SVL, which we used as an index of male body condition. We distinguished males from females by the presence of vocal sacs and shoulder glands in males. Each frog was marked using a numbered waistband and a passive radio frequency identification (RFID) chip (Trovan^®^) implanted under its skin. Any frog without a waistband was captured by hand and processed as above unless it was identified by RFID. Recaptured individuals received a new waistband. 

During our censuses, we used scan sampling to identify potential mating events, which we noted as when a female appeared to be responding to a calling male, for example by sitting or approaching to within close proximity of a male. When such events were identified, we switched to focal sampling and observed the pair until they separated after amplexus or until the female left the area. For all observed amplexus events, we used a watch to record the times (to the nearest 1 s) when the male initially clasped the female, when the female oviposited (i.e., from the first to the last appearance of new eggs), and when the amplectant pair separated. From these data, we were able to compute amplexus duration as well as the durations of the pre-oviposition, oviposition, and post-oviposition periods. Sometimes unmated males interfered with amplectant pairs, causing them to break amplexus. We scored such events as mating failures. For all other amplexus events, in which females completed oviposition, we counted the number of eggs to determine clutch size. We also collected at least 50 eggs (approximately 10 each sampled from the middle of the clutch and from its periphery in the four cardinal directions) to estimate fertilization rate. Eggs from each clutch were transported to a nearby laboratory (50 m away from the study site) where they were placed in a water-filled plastic cup (6.2 cm in radius and 5.1 cm in height). We computed the proportion of collected eggs that developed into embryos within one week as a measure of fertilization rate.

Based on our censuses, we computed nightly OSR (adult males to adult females observed that night) and the population size (total number of adults observed that night). We determined features of territorial residency after completion of our field observations. We operationally defined a territorial residency as when a male occupied and defended a general area (e.g., < 2-3 m^2^) for at least 24 h (i.e., on two consecutive nights of observation). We labelled all the positions of a resident male within a territory and used the minimum convex polygon method [45] to calculate its territory size. After the area of a residential male’s territory was defined, we used the vegetation distribution map to calculate the percentage of its territory area that was covered by submerged grass.

### Data analysis and statistics

We used two-tailed Welch’s *t*-test to compare male and female body sizes and the fertilization rates of undisturbed pairs and pairs that were disturbed by interference from unpaired males, but nevertheless successfully completed oviposition. A χ^2^ test was used to compare the proportion of amplexus events that ended with successful deposition of an egg clutch between pairs that were and were not disturbed. We used two-tailed paired *t*-tests to compare the durations of the pre-oviposition and oviposition periods. We used Spearman's rank correlation to examine the relationship between female SVL and clutch size. 

We used separate generalized linear mixed-effect models (GLMMs) to identify factors that explained variation in the dependent variables of total amplexus duration, the durations of the pre-oviposition and oviposition periods, and fertilization rate. Post-oviposition periods were negligibly short because amplexus ended immediately after the oviposition period, so we did not include this as a dependent variable in our statistical analyses. The independent variables used in these analyses included male SVL, male condition, territory size, percentage of aquatic vegetation coverage in the territory, OSR, population size, and clutch size. We used the adehabitat package [46] and the minimum convex polygon method to calculate territory size. In the analysis of fertilization rate, we additionally included total amplexus duration as an independent variable. For most (75 of 122) of the observed mating events, we lacked information about the female; therefore, we could not test the relationships between female body characteristics and amplexus duration or fertilization rate. We initially included all measured variables to create a “full model” for each dependent variable, and then constructed the “minimal model” by removing terms sequentially using Akaike’s Information Criterion (AIC) [47] until the model contained only terms for which their inclusion significantly reduced the AIC of the model. Male identity was entered as a random effect in these models because this allowed for repeated sampling of the same focal individuals without pseudoreplication [48]. We used restricted maximum likelihood (REML) methods for model estimation. We used the “lmer()” function in the lme4 package to run GLMMs [48]. We set family to “normal” for the analysis of fertilization rate and present *t* values for the final model. We set family to “Poisson” for all other GLMM analyses and present *Z* values for our final models. The significance of the random effect was evaluated using likelihood ratio tests based on comparing models with and without the random effect and we present χ^2^ values for these analyses [49]. 

All analyses were performed using the statistical analysis software R 2.13.1 [50] with an alpha level of 0.05.

### Ethics statement

All procedures were conducted in strict accordance with regulations established by the Experimental Animal Care and Use Committee of Tunghai University. We released all animals at their site of capture within 10 to 30 min of capture. Males usually resumed their activities (e.g., calling) within a few minutes of being released. Over the first two nights of the study, we discovered that females halted their breeding activities after being captured. Thereafter, all the females were captured after they finished mating to minimize disturbance. We made waistbands using waterproof paper tied to a cotton thread, which typically came loose within two weeks. Hence, we could mark individual frogs while reducing the possibility they would be wounded by long-lasting waistbands. For RFID chip injection, each sterile syringe was used only once, and animals received liberal application of antibiotic cream at the site of their insertion wound. We obtained an official permit (TFRILHC0950001568) from the Lien-Hua-Chih Research Center of Taiwan Forestry Research Institute to conduct this study. No other specific permits were required because the field studies were not conducted in a protected area, and the specimens did not involve endangered or protected species.

## Results

During the 64 observation nights, we captured and marked 72 males and 107 females and counted the number of eggs in clutches from 329 amplexus events (Table 1). Measurements of SVL and body mass revealed that males are smaller than females (Table 1; SVL: *t* = -6.18, Welch’s df = 172.5, *P* < 0.001; body mass: *t* = -8.12, Welch’s df = 172.5, *P* < 0.001). However, because of the large overlap between sexes in SVL and body mass, adult size was not a practical characteristic to identify sex (Table 1). We observed an average of 22 ± 9 (mean ± SD here and elsewhere) frogs per night and the average nightly OSR (males/females) was 2.6 ± 1.6 (Table 1), indicating the OSR was typically male biased. Of the 329 amplexus events observed, we were able to observe 122 of these (37%) from before the male and female entered into amplexus until after the pair had successfully oviposited and then separated; these complete, successful amplexus events constitute the data in the GLMM models described below. 

**Table 1:** Sample sizes (*N* = total sample / samples used in GLMM analysis), means, standard deviations (SD), coefficients of variation (CV), and ranges based on the total sample size for male and female body traits, territory traits, nightly population traits, amplexus durations, and clutch size of *Rana adenopleura*.

	Mean ± SD	CV (%)	Range
Amplexus durations (N=122/122)			
Pre-oviposition period (minutes)	7.7 ± 4.5	58.3	1.0 – 27.0
Oviposition period (minutes)	3.2 ± 1.1	33.2	1.3 – 8.7
Total amplexus duration (minutes)	10.9 ± 4.6	41.9	4.0 – 29.5
Egg clutch characters (N=329/122)			
Clutch size	354 ± 90	27.4	60 – 744
Fertilization rate	88.1 ± 22.9	26.0	0 – 100
Male body traits (N=72/31)			
SVL (mm)	51.5 ± 2.7	5.2	42.1 – 58.9
Weight (g)	16.3 ± 2.1	12.7	11.1 – 23.1
Female body traits (N=107/none)			
SVL (mm)	55.2 ± 3.4	6.2	47.4 – 64.7
Weight (g)	18.6 ± 2.7	14.8	12.6 – 26.5
Territory traits (N=78/41)			
Size (m^2^)	0.65 ± 0.78	120.2	0.01 – 4.17
Vegetation coverage (%)	54.3 ± 43.6	80.3	0 – 100
Nightly population traits (N=64/40)			
Sex ratio (♂/♀)	2.6 ± 1.6	56.8	0.78 – 7.3
Population size	21.8 ± 9	34.2	5– 41

### Territoriality, amplexus, and oviposition

**Figure 1:**
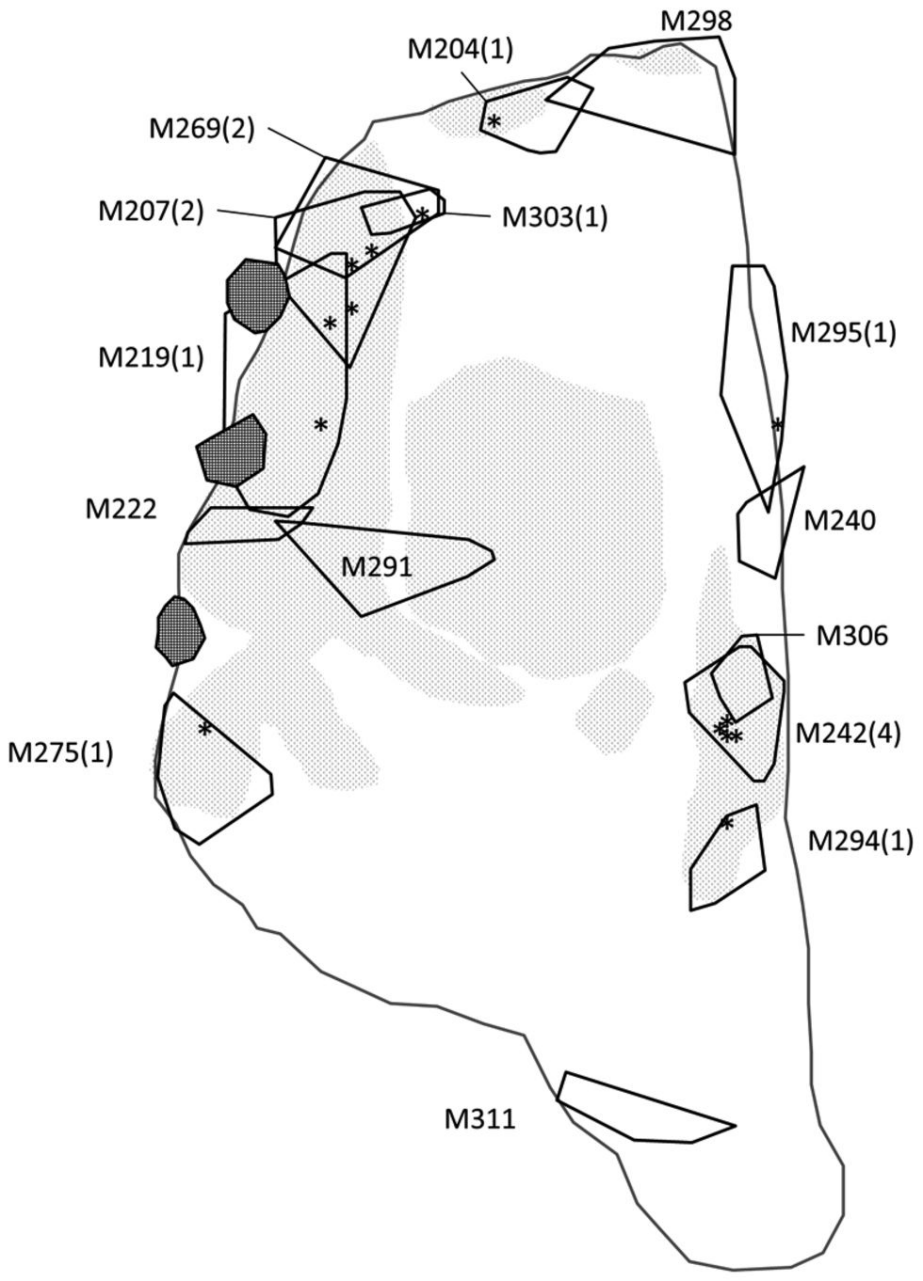
Territories of males that appeared in the study pond on August 22, 2008. There are three stones aside the pond (black areas). A solid black line encloses the estimated territory of each male. Labels represent the ID of a male and the number of egg clutches laid in the territory on that night. Egg clutches laid on that night are indicated by *. Gray areas represent aquatic vegetation measured as part of this study.

During our observations, 26 of 72 males (36%) either never established a territory or defended their calling site for only one observation night (< 24 h). Forty-six of 72 males (64%) established a territory at least once, with an average of 1.7 ± 1.11 different territories for each male (range = 1-5 territories per male). We recorded a total of 78 territory occupancy events, each lasting for an average of 8.4 ± 8.3 nights (range = 2-43 nights). The average territory size was 0.65 ± 0.78 m^2^, and 54.3 ± 43.6% of this area was covered by submerged vegetation on average (Table 1). Most territories were within 0.5 m of the shore in shallow water (< 30 cm depth); none of the territories was established in the deeper water toward the centre of the pond. All observed clutches were deposited inside the territories in which the amplectant male was resident (Figure 1). Females released an average of 354 ± 90 eggs into a single egg mass. Clutch size was positively correlated with female body length (Spearman’s rank correlation, *r*_s_ = 0.408, *P* = 0.002, *N* = 56). The average fertilization rate was 88.1 ± 22.9% (*N* = 329 clutches).

**Figure 2:**
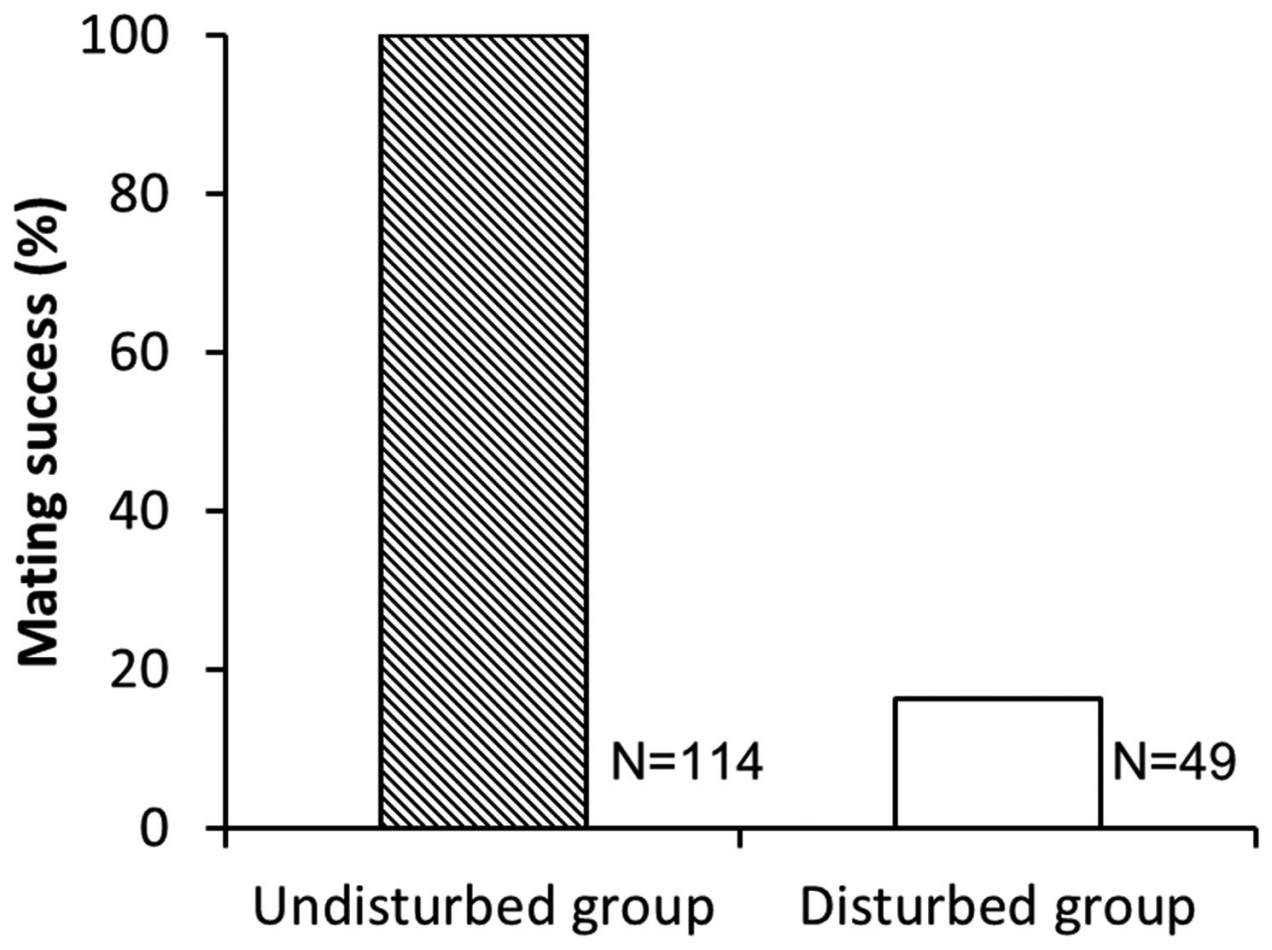
The mating success rate of undisturbed pairs and disturbed pairs. Mating success was defined as successfully completing oviposition before breaking amplexus. The mating success rates were different significantly (χ^2^ = 133.1, df = 3, *P* < 0.001) between undisturbed pairs (100%, *N* = 114) and disturbed pairs (16%, *N* = 49).

When pairs entered amplexus, most males emitted soft, single-note courtship calls. These calls appeared occasionally to attract rivals that subsequently attacked the amplectant pair. In our observations, 114 amplectant pairs oviposited without body-contact interference from intruders, but 49 amplectant pairs were attacked during amplexus. Of these 49 pairs, 41 pairs (84%) terminated amplexus and 8 pairs (16%) successfully repelled the attacker and oviposited (bringing the total number of observed successful amplexus events to 122). The proportion of pairs successfully producing an egg clutch was significantly lower for pairs that were attacked by an unpaired male compared with undisturbed pairs (Figure 2; χ^2^ = 133.1, df=3, *P* < 0.001).

On average, pairs spent about 11 min in amplexus, with no amplexus event lasting longer than 30 min (Table 1). About 70.8% (≈8 min) of the total amplexus duration was spent in the pre-oviposition period. The onset of the oviposition period was characterized by the release of eggs by females. Males regularly squeezed females using their hind legs once females began releasing eggs (see Video S1). On average, males squeezed females 23 ± 3 times (range = 19-29, *N* = 15). The release of eggs often appeared to be concurrent with squeezing by the male. The duration of the oviposition period was significantly shorter and also less variable than the pre-oviposition period (Table 1; paired *t* = 10.69, df = 121, *P* < 0.001). 

### Factors influencing amplexus duration

In our GLMM analyses of amplexus duration, male SVL, male body condition, territory size, vegetation coverage, and population size had no effects in the full model (AIC = 201.9) and were subsequently removed to create the minimal model (Table 2). In the minimal model (AIC = 197.3), amplexus duration was positively related to OSR and clutch size (Table 2). The effects of OSR and clutch size on amplexus duration were largely due to their influence on the duration of the pre-oviposition period. In separate analyses of the pre-oviposition period, male SVL, male body condition, territory size, vegetation coverage, and population size were removed from the full model (AIC = 252.8) to create the minimal model (Table 3). In the minimal model (AIC = 250.1), the pre-oviposition period was positively related to both OSR and clutch size (Table 3). In analyses of the oviposition period (Table 4), all of the variables entered were subsequently removed from the full model (AIC = 53.8) and no variable was related to the length of the oviposition period in the minimal model (AIC = 43.1). The duration of the pre-oviposition period, but not total amplexus duration and the duration of the oviposition period, differed significantly among different individual males (random factor, Tables 2, 3, and 4). 

**Table 2:** Results of the GLMM model for total amplexus duration for *Rana adenopleura*.

Factor	Full model (AIC = 201.9)				Minimal model (AIC = 197.3)			
	Estimate	SE	*Z* or χ^2^	*P*	Estimate	SE	*Z* or χ^2^	*P*
Male identity (random factor)	0.015	0.123	1.06	0.095	0.020	0.142	1.06	0.095
Intercept	1.68	0.86	1.95	0.051	2.01	0.14	14.09	< 0.001***
Clutch size	0.0008	0.0003	2.35	0.019*	0.0007	0.0003	2.23	0.026*
OSR	0.09	0.03	3.02	0.003**	0.06	0.03	2.27	0.023*
Population size	0.006	0.004	1.28	0.199				
Territory size	0.08	0.05	1.53	0.126				
Vegetation coverage	-0.001	0.001	-1.03	0.304				
Male SVL	0.001	0.016	0.084	0.933				
Male condition	-0.04	0.03	-1.16	0.246				

*P* < 0.05^*^

** *P*<0.01

*** *P* < 0.001

**Table 3:** Results of the GLMM model for the pre-oviposition period of amplexus in *Rana adenopleura*.

Factor	Full model (AIC = 252.8)				Minimal model (AIC = 250.1)			
	Estimate	SE	*Z* or χ^2^	*P*	Estimate	SE	*Z* or χ^2^	*P*
Male identity (random factor)	0.054	0.233	2.20	0.04*	0.054	0.233	2.20	0.04*
Intercept	1.09	1.27	0.86	0.39	1.50	0.18	8.45	< 0.001***
Clutch size	0.001	0.0004	2.63	0.008**	0.001	0.0004	2.58	0.01*
OSR	0.11	0.04	2.99	0.003**	0.08	0.03	2.22	0.03*
Population size	0.007	0.005	1.28	0.20				
Territory size	0.13	0.07	1.78	0.07				
Vegetation coverage	-0.002	0.001	-1.335	0.18				
Male SVL	0.002	0.024	0.095	0.92				
Male condition	-0.07	0.05	-1.43	0.15				

* *P* < 0.05

** *P* < 0.01

*** *P* < 0.001

**Table 4:** Results of the GLMM model for the oviposition period of amplexus in *Rana adenopleura*.

Factor	Full model (AIC = 53.78)				Minimal model (AIC = 43.14)			
	Estimate	SE	*Z* or χ^2^	*P*	Estimate	SE	*Z* or χ^2^	*P*
Male identity (random factor)	0	0	-2.85	1.00	0.00	0.00	-2.85	1.00
Intercept	1.81	0.30	1.39	0.16	1.16	0.05	22.97	< 0.001***
Clutch size	3.63E-05	6.22E-04	0.06	0.95				
OSR	4.91E-02	5.18E-02	0.95	0.34				
Population size	5.03E-03	7.58E-03	0.66	0.51				
Territory size	-6.18E-02	7.83E-02	-0.79	0.43				
Vegetation coverage	7.04E-04	1.71E-03	0.41	0.68				
Male SVL	-1.70E-02	2.42E-02	-0.70	0.48				
Male condition	1.84E-02	4.88E-02	0.38	0.71				

*** *P* < 0.001

### Factors influencing fertilization rate

In our GLMM analyses of factors affecting fertilization rate (Table 5), none of the variables entered into the full model (AIC = 55.4) exhibited a significant relationship with fertilization rate. However, the effects of amplexus duration (*P* = 0.08) and male SVL (*P* = 0.12) both exhibited interesting trends in opposite directions (Table 5). Variables from the full model were removed sequentially based on their having the lowest *Z* value until all remaining variables were significant. In the reduced minimal model (AIC = 43.1), there was a significant negative relationship between fertilization rate and amplexus duration and a significant positive relationship between fertilization rate and male SVL (Table 5). Recall that 84% of attacks (41 of 49) of amplectant pairs by unpaired males ended with the separation of the amplectant pair prior to oviposition (Figure 2). In the 8 pairs (16%) that successfully repelled attacks and mated, the fertilization rate (80.1 ± 12.1%, *N* = 8) was lower, though not significantly so, in comparison with the fertilization rates of undisturbed pairs (90.6 ± 1.7%, *N* = 114) (*t* = -0.86, Welch’s df = 7.27, *P* = 0.42).

**Table 5:** Results of the GLMM model for fertilization rate in *Rana adenopleura*.

Factor	Full model (AIC = 55.4)				Minimal model (AIC = 43.1)			
	Estimate	SE	*t* or χ^2^	*P*	Estimate	SE	*t* or χ^2^	*P*
Male identity (random factor)	350.35	18.72	-2.94	0.09	349.10	18.68	-2.94	0.086
Intercept	25.56	46.38	0.55	0.59	15.02	38.23	0.39	0.697
Clutch size	5.75E-03	0.02	0.27	0.79				
OSR	-1.39	1.90	-0.74	0.47				
Population size	0.08	0.26	0.32	0.75				
Territory size	-3.74	2.80	-1.34	0.19				
Vegetation coverage	0.03	0.06	0.50	0.62				
Male SVL	1.40	0.87	1.61	0.12	1.63	0.74	2.20	0.036*
Male condition	0.82	1.83	0.45	0.66				
Amplexus duration	-0.72	0.40	-1.83	0.08	-0.85	0.37	-2.29	0.029*

* *P* < 0.05

## Discussion

### Amplexus duration compared with other frogs

Although amplexus durations range widely within the anuran order (reviewed in 33,35), *Rana adenopleura* is notable for having a relatively short (11 min) amplexus duration compared with other frogs [34–36,38,42,51–55], which range from 37 mins in *Limnonectes kuhlii* (formerly *Rana kuhlii*) [35] to more than one month in *Atelopus varius* [56]. For many frogs, the pre-oviposition period is the most important component explaining variation in amplexus duration [34,57]. In the present study, the pre-oviposition period in *R. adenopleura* made up 70.8% of the total amplexus duration, which is similar to findings of earlier studies in other frog species. For example, the pre-oviposition period of *R. arvalis*, during which pairs move to an oviposition site, lasts 6.8 hr (81.5 % of the amplexus duration) [51]. Similarly, it takes 3.8 – 31.6 hr (16.9 hr on average, 80% of the amplexus duration) for *R. torrenticola* to find suitable oviposition sites [34].

 Compared to the pre-oviposition period, the actual oviposition period is shorter and less variable in *R. adenopleura*, which is consistent with reports of amplexus in two congeners from North America, the bullfrog, *R. catesbeiana*, and the green frog, *R. clamitans* [33,58]. The relatively short oviposition period in these species may result from the fact that eggs are laid in a single bout. In species that lay eggs in series of separate bouts, amplectant pairs often spend time searching for, or moving between, different oviposition sites, so the oviposition period takes longer and varies more depending on the intervals of oviposition. For example, *Buergeria japonica* lays a single bout of eggs in less than 1 min; however, the total duration of two observed amplexus events lasted between about 50 min (14 egg laying bouts, 19 movements) and one hr (nine egg laying bouts, 14 movements) [53]. Another reason for the short oviposition period in *R. adenopleura* is that eggs are laid in a mass without further modification (e.g., requiring no nest). Wells [33] reviewed the relationship between the type of egg mass and oviposition time across some 40 frog species with axillary amplexus. Oviposition time is usually much longer when eggs are oviposited in long strings of eggs and in foam nests. Finally, amplectant pairs of *R. adenopleura* separated immediately after oviposition; hence, there was no post-oviposition period in this species. The function of post-oviposition amplexus could be insurance of fertilization [33–35]. Post-oviposition amplexus is reported in species that lay eggs in multiple bouts [35] or in long strings [34]. Compared with other anuran species, amplexus duration in *R. adenopleura* is short, at least in part, because all eggs are oviposited in a single, large mass within the chosen males’ territory. This finding is consistent with reports in species with similar modes of oviposition [33,58].

### Factors influencing amplexus duration

Here, we have shown that amplexus duration in *R. adenopleura* (Table 1), in particular the duration of the pre-oviposition period (Table 2), was directly related to the magnitude of nightly male-bias in the OSR. Positive relationships between mating duration and male-biased sex ratios have also been reported in many internally fertilizing insects [15,16,20–22]. The usual adaptive explanation is that prolonged mating in internally fertilizing animals acts as a form of post-insemination mate guarding to prevent re-insemination of females and to increase sperm loading under intense sperm competition [9,15]. This adaptive function, however, is not particularly well suited for externally fertilizing animals (but see 59,60) and cannot explain our findings. Because female *R. adenopleura* deposit their eggs in one bout in a relatively short period of time, and males fertilize eggs upon their release, mate guarding is not necessary to prevent females from re-mating or to decrease the intensity of sperm competition. We believe the direct relationship between the duration of the pre-oviposition period (and hence amplexus duration) and the degree of male bias in the nightly OSR reflects the effects of interference on amplectant pairs by unpaired males. It is well established that as the OSR becomes more male biased, the potential for male-male competition also increases [1,62]. During the study period, amplectant males responded vocally to the encroachment and vocalizations of intruding males and nearby residential males (Chuang, per obs). If vocal threats did not repel the intruding males, amplectant males were attacked (49 cases). Those instances of contact (by body) and non-contact (by visual or acoustic) interference likely prolonged the amplexus duration by increasing the duration of the pre-oviposition period. Such effects also have been reported in other animals [6,35,61]. 

We also found that amplexus duration was positively related to clutch size, which is consistent with findings from previous studies [19,28,29]. However, in contrast to what might have been expected, we found that clutch size was related to the duration of the pre-oviposition period but not the actual oviposition period itself. Thus, the positive relationship between clutch size and amplexus duration does not result simply because females require more time to lay larger clutches. Two explanations for this result are possible. First, size differences between particularly large females (which have more eggs) and small males might impose biomechanical constraints related to forming a stable amplexus posture [63]. However, our preliminary data revealed no significant relationship between amplexus duration and the size difference between males and females in amplexus (Spearman’s rank correlation, *r* = -0.16, *N* = 55, *P* = 0.25, Chuang, M-F unpublished data), suggesting biomechanical constraints are an unlikely explanation for the positive relationship between clutch size and the pre-oviposition period reported here. A second explanation is related to the time required for egg transfer to the oviducts during amplexus. In some ranid frogs, eggs do not begin moving from the ovaries into the oviducts until the female enters amplexus [33]. Hence, time to move all of the eggs into the oviducts may increase with clutch size, which could explain the positive correlation between clutch size and pre-oviposition period in *R. adenopleura*.

Territory size and male body size and condition were not related to amplexus duration in our study. In species with resource defence mating systems, males defend territories that provide critical resources for reproduction, including mating and oviposition sites [33,64]. We found that all clutches were located in males’ territories (Figure 1), indicating that territories provide oviposition sites for females in *R. adenopleura*. Thus, time spent searching for a suitable oviposition site while in amplexus is greatly reduced, which translates into no relationship between amplexus duration and territory traits. On the other hand, the lack of any relationship between male physical traits (including SVL and body condition) and amplexus duration may be due to the small variation observed in these traits in our study (coefficient of variations in male SVL and condition were 5.2% and 12.7%, respectively, Table 1). Some earlier studies failed to find effects of male body size on female mate selection because females were presumably unable to distinguish between small and large males due to small variation in male body size [65,66]. 

### Factors influencing mating success and fertilization rate

Our results showed that the aggressive interactions between amplectant and unpaired males could lead to the termination of amplexus events or the reduction of fertilization rate if amplectant pairs successfully repelled the interfering male. In this study, the OSR varied by nearly an order of magnitude across nights, ranging between 0.78 – 7.3, and was more often male biased. We observed 49 out of 114 amplexus events with contact interference by an unpaired male, indicating a high rate (30%) of contact mating interference. Among these instances of interference, the vast majority (41/49 cases) resulted in termination of amplexus without egg deposition (Figure 2). The relatively high rate of contact interference, combined with the low rate of success defending against interfering males, indicates that direct male-male competition can have significant costs to amplectant pairs in this species in terms of lost mating opportunities. From the male perspective, the loss of a mating opportunity, after successfully attracting a female, could significantly impact lifetime reproductive success. From the perspective of the female, the loss of a mating opportunity might result in her eventual pairing with a less preferred male or perhaps even the eventual loss of a clutch. In addition, there is a potential cost associated with risk of reduced fertilization success as a result of non-contact interference. For example, in one observed case of interference, an amplectant male reversed his body position atop the female to face the intruding male, ultimately causing none of the eggs in the clutch to be fertilized (Chuang, per obs).

For those males that successfully mated with females and deposited eggs, our study provides some evidence that the factors under consideration here influenced fertilization rate. In the full GLMM model we considered, none of the factors were able to predict fertilization rate. In the minimal model, however, fertilization rate of egg clutch was negatively related with amplexus duration but positively related with male body size. The observed positive relationship between male body size and fertilization rate is relatively straightforward and indicates larger males were able to fertilize a higher proportion of the eggs in a female’s clutch. This relationship could result because smaller males were sperm limited [67], or perhaps because larger males were better able to deter or defend against interference by smaller unpaired rivals [68]. These two explanations are not mutually exclusive. However, the observed negative relationship between amplexus duration and fertilization rate is perhaps less intuitive in that it indicates *longer* amplexus durations were actually associated with *lower* fertilization rates, not higher fertilization rates. We suggest the following explanation. Recall that aggressive interactions between amplectant and unpaired males were common and that one of the primary determinants of amplexus duration was the nightly male bias in the OSR. Attacks by unpaired males were highly successful in disrupting mating by amplectant pairs causing mating failure (Figure 2), and fertilization rates were somewhat lower in the few disturbed pairs that successfully repelled attackers. It seems likely that efforts by amplectant males to repel attacks from unpaired males may have resulted in both prolonged amplexus and a reduction in the number of eggs fertilized. Although OSR did not enter into the minimal model as having a significant influence on fertilization rate, there was nevertheless a negative relationship between fertilization rate and both amplexus and OSR in the full model (Table 5). While the linkage between male bias in the OSR and fertilization rate is indirect, it nevertheless seems plausible that costs associated with intense male-male competition might include not only lower mating success but also reduced fertilization success.

## Conclusions

We described the temporal aspects of amplexus, identified potential casual factors influencing variability in amplexus duration, mating success, and fertilization rate, and discussed possible adaptive explanations for this variability in an anuran with a resource defence mating system. To our knowledge, this is the first study of causal factors underlying variation in mating duration in an externally fertilizing species, and it is one of the few such studies in vertebrate animals. The amplexus duration of about 11 min in *R. adenopleura* is, so far, the shortest ever described for anurans. This brief amplexus duration, together with the positive relationship between amplexus duration and male-bias in the OSR and the negative relationship between amplexus duration and fertilization rate, suggest that intense male-male competition may act as a source of selection in the evolution of reproductive behaviour in this species. Interference from unpaired conspecific males results in increased reproductive costs that potentially include time and energy wasted, mating failure, and lower fertilization rates. We suggest that the relatively short mating duration in *R. adenopleura* is likely selected to reduce these potential costs. Additional manipulative experiments will be necessary to further evaluate unexplained findings (e.g., the positive relationship between clutch size and the duration of the pre-oviposition period but not the oviposition period) and to evaluate causes underlying variability in mating duration. More empirical evidence is also needed to evaluate the effects of male body traits and territory traits on mating duration in these territorial animals. Comparative studies would help identify the influence of different mating systems on the evolution of mating duration.

## Supporting Information

Video S1 **The oviposition behaviour of *Rana adenopleura*.** In amplexus, the release of eggs by the female often appeared to be concurrent with synchronous squeezing by the male.
(MP4)
